# Selenium-Containing Protein From Selenium-Enriched *Spirulina platensis* Attenuates High Glucose-Induced Calcification of MOVAS Cells by Inhibiting ROS-Mediated DNA Damage and Regulating MAPK and PI3K/AKT Pathways

**DOI:** 10.3389/fphys.2020.00791

**Published:** 2020-07-09

**Authors:** Cong Lin, Li-jun Zhang, Bo Li, Feng Zhang, Qing-rong Shen, Guo-qing Kong, Xiao-fan Wang, Shou-hong Cui, Rong Dai, Wen-qiang Cao, Pu Zhang

**Affiliations:** ^1^Department of Cardiology, The Second Affiliated Hospital of Wenzhou Medical University, Wenzhou, China; ^2^Department of Neurology, People’s Hospital of Linyi Affiliated to Qingdao University, Linyi, China; ^3^Department of Emergency, Taian City Central Hospital, Taian, China; ^4^Physical Examination Center, Taian City Central Hospital, Taian, China; ^5^Department of Biotechnology, Zhuhai Hopegenes Medical and Phamaceutical Institute, Zhuhai, China; ^6^Department of Cardiovascular Medicine, Taian City Central Hospital, Taian, China

**Keywords:** hyperglycemia, vascular calcification, cardiovascular and cerebrovascular diseases, Se-containing protein, *Spirulina platensis*, ROS

## Abstract

Hyperglycemia is the main feature of diabetes and may increase the risk of vascular calcification (VC), which is an independent predictor for cardiovascular and cerebrovascular diseases (CCD). Selenium (Se) may decrease the risk of CCD, and previous studies confirmed that Se-containing protein from Se-enriched *Spirulina* platensis (Se-SP) exhibited novel antioxidant potential. However, the effect of Se-SP against VC has been not investigated. Herein, the protective effect and underlying mechanism of Se-SP against high glucose-induced calcification in mouse aortic vascular smooth muscle cells (MOVAS) were explored. Inductively coupled plasma atomic emission spectroscopy (ICP-AES) results showed time-dependent uptake of Se-SP in MOVAS cells, which significantly inhibited high glucose-induced abnormal proliferation. Se-SP co-treatment also effectively attenuated high glucose-induced calcification of MOVAS cells, followed by decreased activity and expression of alkaline phosphatase (ALP). Further investigation revealed that Se-SP markedly prevented reactive oxygen species (ROS)-mediated DNA damage in glucose-treated MOVAS cells. ROS inhibition by glutathione (GSH) effectively inhibited high glucose-induced calcification, indicating that Se-SP could act as ROS inhibitor to inhibit high glucose-induced DNA damage and calcification. Moreover, Se-SP dramatically attenuated high glucose-induced dysfunction of mitogen-activated protein kinases (MAPKs) and phosphatidylinositol-3-kinase/AKT (PI3K/AKT) pathways. Se-SP after Se addition achieved enhanced potential in inhibiting high glucose-induced calcification, which validated that Se-SP as a new Se species could be a highly effective treatment for human CCD.

## Graphical Abstract

Se-containing protein from Se-enriched *Spirulina* platensis (Se-SP) was extracted and characterized. Se-SP *in vitro* effectively attenuated high glucose-induced abnormal proliferation and calcification of MOVAS cells through inhibiting ROS-mediated DNA damage and regulating MAPKs and PI3K/AKT pathways.

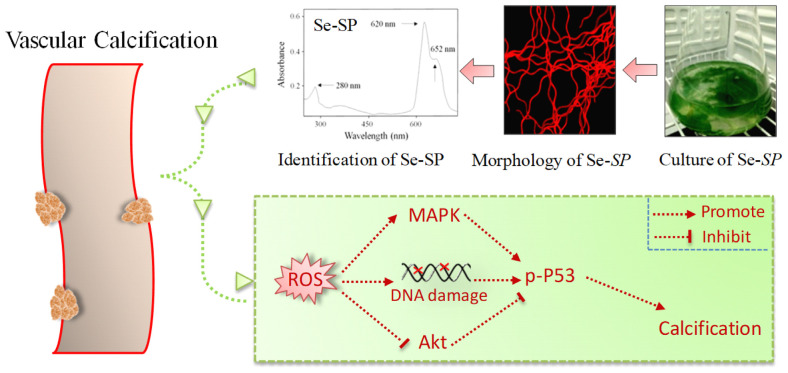

## Introduction

Diabetes is a metabolic disease characterized by high glucose and hyperinsulinemia that has reached epidemic proportions (Ogurtsova et al., 2017). As a consequence of modern life styles, the global prevalence of diabetes is increasing (Cosentino et al., 2020). Vascular calcification (VC) is a common complication of diabetes and is an indicator of atherosclerosis (Berliner et al., 1995). VC may cause arterial stiffness, luminal stenosis, and plaque instability (Wu et al., 2016), which was an independent risk factor for morbidity and mortality of cardiovascular and cerebrovascular diseases (CCD) (Bugnicourt et al., 2011; Leon and Maddox, 2015). VC could also limit complete expansion of a stent or balloon during interventional therapy (He et al., 2019). VC was also associated with poor prognosis after revascularization (Lee et al., 2015). Inhibition of VC represents a novel way to treat human CDD in clinic.

Vascular smooth muscle cells (VSMCs) are the primary cytological basis for VC, which is similar to bone formation. Expression of bone-specific molecules such as runt-related transcription factor 2 (Runx2), bone morphogenetic protein 2 (BMP2), alkaline phosphatase (ALP), and type I collagen (Col I) is highly upregulated in calcified VSMCs (Liu et al., 2013). Many studies have confirmed that abnormal VSMC migration, proliferation, and apoptosis all contributed to VC pathogenesis (Byon et al., 2008; Durham et al., 2018). Epidemiological investigation showed that chronic hyperglycemia in diabetic patients significantly increased the risk of VC. High glucose *in vitro* also affected the migration, proliferation, apoptosis, and calcification of VSMCs through regulating mitogen-activated protein kinases (MAPKs) (Shi et al., 2017). Additionally, oxidative stress can promote VC pathogenesis by regulating Runx2 and phosphatidylinositol-3-kinase (PI3K/AKT) signaling pathways (Byon et al., 2008). Similarly, high glucose can induce overproduction of reactive oxygen species (ROS) that promote the proliferation and VC of VSMCs by regulating MAPK and PI3K/AKT signaling (Li et al., 2013). Accumulating evidence indicates that constant hyperglycemia in diabetes could induce ROS overproduction through multiple mechanisms (Giacco and Brownlee, 2010; Pickering et al., 2018; Mendoza-Nunez et al., 2019). Excessive ROS can impair the endogenous antioxidant system, cause redox imbalance, and eventually induce VC in VSMCs (Matough et al., 2012). However, the underlying mechanism remains unclear.

Selenium (Se) is an essential micronutrient for human health with multiple advantageous biological properties, such as antioxidant and antitumor activities and immune regulation (Huang et al., 2012; Ruggeri et al., 2019; Xia et al., 2019). Inorganic and organic Se usually induces severe toxicity. However, Se was metabolized in the biological environment and ultimately incorporated into non-toxic Se-containing proteins. Several antioxidants including glutathione (GSH) peroxidase and thioredoxin reductase contain Se-active domains, which play key roles in regulating redox signaling (Rayman, 2000). *Spirulina platensis* was rich in essential amino acids, fatty acids, vitamins, and other nutritional substances, which was accepted as the most nutrient-enriched functional food (Hosseini et al., 2013). Many studies have confirmed that *S. platensis* showed anti-oxidative, anti-inflammatory, and immune stimulatory properties (Abdel-Daim et al., 2018, 2019, 2020; Aladaileh et al., 2020). However, little information about Se-containing *S. platensis* was available. We previously demonstrated that *S. platensis* is a suitable Se carrier, and Se incorporation increased inhibition of oxidative stress in human diseases (Chen et al., 2006b; Zhang et al., 2011; Fan et al., 2012). Se can attenuate VSMC calcification by suppressing oxidative stress (Liu et al., 2010, 2014), but the potential of Se-containing protein against VC has been not reported. In this study, high glucose was employed to establish a VC model of diabetes in mouse aortic vascular smooth muscle cells (MOVAS). The protective effects and mechanism of Se-containing protein from Se-enriched *S. platensis* (Se-SP) against high glucose-induced calcification of MOVAS cells were explored.

## Experimental Section

### Materials

2,7-Dichlorofluorescein diacetate (DCFH-DA), 3-(4,5-dimethylthiazol-2-yl)-2,5-diphenyltetrazolium bromide (MTT), alizarin red, and ALP staining and activity kits were obtained from Beyotime (Shanghai, China). All primary and secondary antibodies and inhibitors were purchased from CST (Danvers, MA, United States). All chemicals and other agents were purchased from Sigma (St. Louis, MO, United States).

### Culture of Se-Enriched *S. platensis*

Se-enriched *S. platensis* was cultured by a stepwise Se addition method as previously reported (Chen et al., 2006b). Briefly, *S. platensis* was cultured with Zarrouk medium in Erlenmeyer flasks at 30°C under a 14:10 h light: dark cycle. Sodium selenite (Na_2_SeO_3_) was added into the medium at dosages of 100 mg/l (day-7), 150 mg/l (day-8), and 200 mg/l (day-9). The total Se dosage was 450 mg/l. *S. platensis* cultured with no Na_2_SeO_3_ was used as the negative control. Se-SP morphology was observed by light and fluorescent microscopy (magnification, 200×).

### Extraction and Characterization of Se-SP

Se-containing protein from Se-SP was extracted by ultrasonication as previously reported (Sun et al., 2018). Briefly, Se-SP cells were collected by filtration, suspended in 50 mM phosphate-buffered saline (PBS, pH7.0), frozen (at −20°C), and thawed (at 4°C) repeatedly 10 times. Then, *S. platensis* cells were treated by ultrasonication for 3 min (Sonics VCX 600 system, 200 W). The supernatant containing crude Se-PC was centrifuged at 11,000 *g* for 30 min. Se-SP crude levels were quantified with bicinchoninic acid (BCA) kits and stored at −80°C for further usage. Absorbance and emission spectra of Se-SP were detected on an ultraviolet and visible spectrophotometer and fluorescence microreader, respectively.

### Cell Culture and Se Uptake

MOVAS cells from American Type Culture Collection (ATCC, Manassas, VA, United States) were cultured with complete Dulbecco’s minimum essential medium containing 5 mM glucose at 37°C and 5% CO_2_. Glucose (final concentration 10–50 mM) was added into the medium. Intracellular uptake of Se-SP was detected by measuring Se content in MOVAS cells using an inductively coupled plasma atomic emission spectroscopy (ICP-AES) method as previously reported (Chen et al., 2006a). Briefly, MOVAS cells seeded in 9-cm dishes were exposed to 20 μg/ml Se-SP for 0–48 h. Cells were washed with PBS, collected by centrifuge, and re-suspended at a density of 10^7^ cells per sample. Then, all the samples were digested with concentrated nitric acid (3 ml) and H_2_O_2_ (1 ml) at 180°C for 3 h, and diluted to 10 ml with Milli-Q water. Se content was examined with the ICP-AES method. Se content was calculated and is presented as μg/10^7^ cells.

### Drug Treatment and Cell Viability

Se-SP (or SP) and/or 10–50 mM glucose for 48 h. Cell viability was detected by MTT assay (Denizot and Lang, 1986). Briefly, treated cells were incubated with 20 μl MTT solution (5 mg/ml in PBS) for 5h at 37°C. Then, the supernatant was gently removed and added to150 μl/well of dimethyl sulfoxide. The culture plate was gently shaken for 5 min before cell viability was measured by detecting the absorbance at 570 nm. The values are presented as % of control.

### Alizarin Red Staining and Measurement of Calcium Content

MOVAS cells seeded in a six-well plate were treated with 10 μg/ml Se-SP (or SP) and/or 25 mM glucose for 14 days. After calcification induction, cells in 6-well culture plates were washed with PBS and subsequently fixed with 10% formaldehyde for 10 min. They were then washed with PBS and stained with 0.1% alizarin red for 20 min at room temperature. Calcium nodules were identified using an inverted phase contrast microscope. Calcium content was semi-quantified by detecting the absorbance of alizarin red. Briefly, alizarin red in calcified cells was eluted with 10% formic acid. The absorbance of alizarin red from calcified cells was detected at 420 nm by a microplate reader, which was indirectly used to reflect the calcium content. Calcified cells were lyzed, and total protein samples were prepared and quantified by BCA kits. Calcium content was detected by the o-cresolphthalein complex method (Qiu et al., 2017). Calcium content was calculated and is expressed as μg/mg protein.

### ALP Staining and Measurement of ALP Activity

Alkaline phosphatase, a marker of cell calcification level, was assayed as previously described (Satomi-Kobayashi et al., 2012). After treatment with Se-SP and/or glucose for 14 days, MOVAS cells were washed with PBS and fixed in 4% paraformaldehyde for 20 min. Then the ALP staining kit was used according to the manufacturer’s instruction (magnification, 200×). Total protein was prepared and quantified by BCA kits. ALP activity in total protein extracts from MOVAS cells was measured at 405 nm using ALP activity kits.

### Measurement of ROS

Early ROS generation in MOVAS cells was measured by a DCFH-DA probe as previously reported (Chen and Wong, 2009). As a non-polar compound, DCFH-DA can rapidly penetrate into cells and be hydrolyzed into the reduced fluorescent polar derivative DCF. ROS can oxidize DCFH to fluorescent DCF, which can be used to quantify ROS generation. Briefly, MOVAS cells seeded in six-well plates were pre-incubated with DCFH-DA for 15 min in dark and treated with 25 mM glucose for 10–120 min. After incubation, cells were washed with PBS, and ROS generation (green fluorescence) was monitored under a fluorescent microscope (magnification, 100×).

### Western Blotting

MOVAS cells seeded in 9-cm dishes were treated with 10 μg/ml Se-SP and/or 25 mM glucose for 14 days. After treatment, cells were collected and lyzed in radioimmuno precipitation assay (RIPA) lysis buffer. Total protein was obtained by centrifugation and quantified with BCA kits. Protein expression was examined by western blotting. Briefly, equal amounts of total protein from each sample were separated by sodium dodecylsulfate-polyacrylamide gel electrophoresis and electrically transferred to nitrocellulose membranes. Membranes were blocked with 5% non-fat milk in Tris buffered saline and Tween-20 for 1 h at room temperature, followed by designated primary antibodies overnight at 4°C. Afterward, membranes were incubated with the appropriate secondary antibodies conjugated with horseradish peroxidase for 2 h at room temperature. Enhanced chemiluminescent substrate was used to detect the protein signal. β-actin expression served as the internal reference.

### Statistical Analysis

All data are expressed as mean ± standard deviation (SD). All experiments were based on at least three independent experiments. Statistical analyses were performed using SPSS 13.0 software (SPSS Inc., Armonk, NY, United States). Differences between two groups were analyzed by two-tailed Student’s *t*-tests, and those among three or more groups were compared with one-way analysis of variance followed by Tukey’s *post hoc* tests. Bars with “*” or “**” indicate a statistical level of *P* < 0.05 and *P* < 0.01, respectively. Bars with different signs are statistically significance at the level of *P* < 0.05.

## Results

### Characterization and Cellular Uptake of Se-SP

**FIGURE 1:**
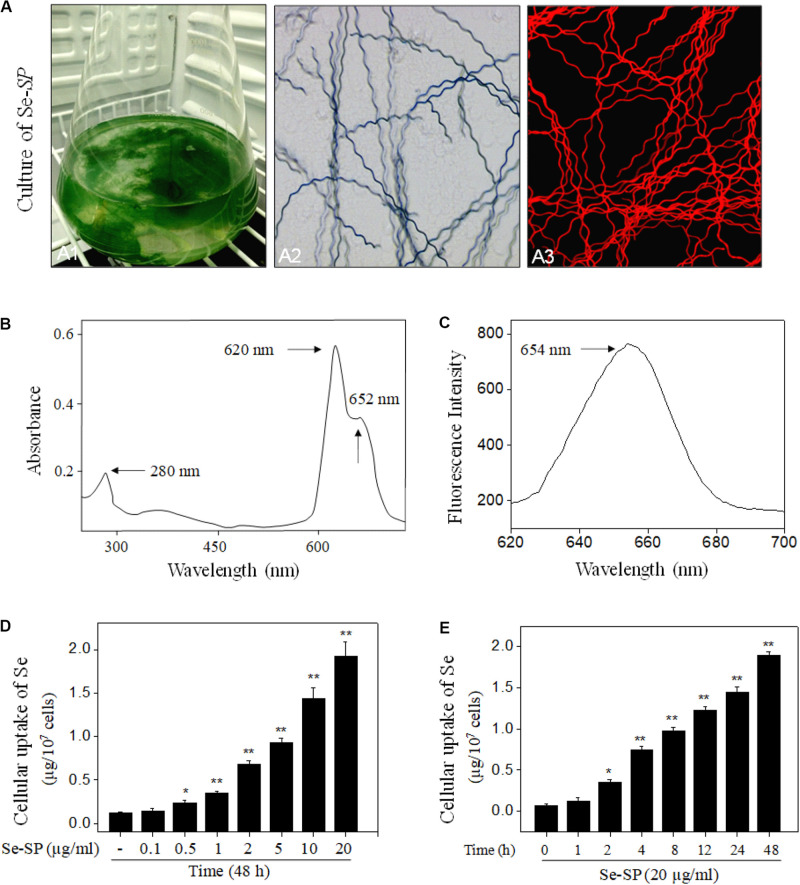
Characterization and intracellular uptake of Se-SP. **(A)** Culture and morphology of Se-enriched *S. platensis.* Se-enriched *S. platensis* was cultured with Zarrouk medium (pH 9.0) in a 1000 ml Erlenmeyer flask **(A1)**. Morphology of Se-enriched *S. platensis* was detected by Light microscope **(A2)** and fluorescence microscope **(A3)**. **(B)** UV–VIS spectra of Se-SP. Se-SP crude was extracted from Se-enriched *S. platensis* by ultrasonication, and absorption spectra of Se-SP were detected by UV–VIS spectrophotometer. **(C)** Emission spectrum of Se-SP. Fluorescence spectrum of Se-SP was examined by fluorescence microreader. Dose-dependent **(D)** and time-dependent **(E)** cellular uptake of Se-PC. MOVAS cells were treated with 0.1–20 μg/ml Se-SP for 1–48 h, and the intracellular uptake of Se-SP was examined by ICP-AES method. All data were shown from three different experiments. Bars with “*” or “**” indicate a statistical level of *P* < 0.05 and *P* < 0.01, respectively. Bars with different letters suggest significance at the level of *P* < 0.05.

*Spirulina platensis* or Se-SP was cultured with Zarrouk medium in Erlenmeyer flask (Figure 1A1) as we previously reported (Minkova et al., 2003; Fan et al., 2012). Morphological observation showed that Se-SP and *S. platensis* both showed the same spiral shape (Figure 1A2), indicating that Se addition did not affect the appearance. Because of the presence of allophycocyanin (APC), Se-SP showed bright red fluorescence under microscopy (Figure 1A3). Importantly, Se-SP was also characterized based on the absorption and fluorescence spectra. As shown in Figure 1B, the specific absorption peaks of phycocyanin (PC) and APC were monitored at 620 and 652 nm, respectively. Se-SP also showed an obvious absorption peak of protein at 280 nm. Moreover, Se-SP under 544 nm excitation showed an obvious emission peak at 654 nm (Figure 1C). These spectral characteristics were consistent with previous studies (Minkova et al., 2003; Chen et al., 2006a; Fan et al., 2012). PC and APC are the main components of Se-SP, and both have large molecular weights (Chen et al., 2006a; Sun et al., 2018). Successful penetration and accumulation of Se-SP in MOVAS cells are key processes for biological effects. Hence, intracellular Se uptake of Se-SP in MOVAS cells was examined with ICP-AES. The results revealed dose- and time-dependent cellular uptake of Se (Figures 1D,E), which indicated that Se-SP successfully accumulated in MOVAS cells.

### Se-PC Inhibits High Glucose-Induced Proliferation of MOVAS Cells

**FIGURE 2:**
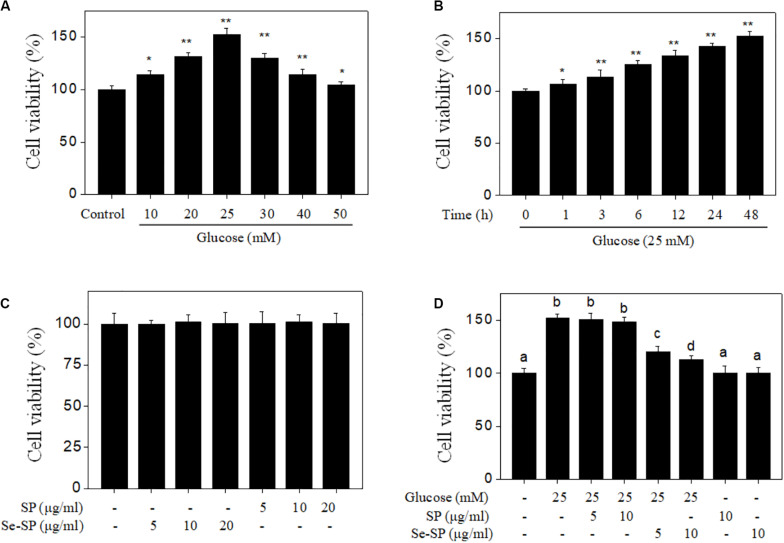
Se-SP inhibits high glucose-induced proliferation of MOVAS cells. **(A)** Dose-dependent proliferation of high glucose-treated MOVAS cells. MOVAS cells were treated with 10–50 mM glucose for 48 h. Cells treated with medium containing 5 mM glucose was set as the control group. **(B)** Time-dependent proliferation of high glucose-treated MOVAS cells. MOVAS cells were treated with 25 mM glucose for 1–48 h. **(C)** Cytotoxicity of SP and Se-SP on MOVAS cells. MOVAS cells were treated with 5–20 μg/ml SP or Se-SP for 48 h. **(D)** Se-SP inhibited high glucose-induced cell proliferation. MOVAS cells were co-treated with 5–10 μg/ml Se-SP and 25 mM glucose for 48 h. Cell viability was detected by MTT assay. All data are shown from three different experiments. Bars with “*” or “**” indicate a statistical level of *P* < 0.05 and *P* < 0.01, respectively. Bars with different letters are statistical significance at *P* < 0.05 level.

Abnormal VSMC proliferation plays an important role in the process of diabetic VC (Chen N. X. et al., 2006; He et al., 2012). Hence, cell proliferation was first screened with MTT assays. As shown in Figure 2A, glucose treatment for 48 h did not cause significant cytotoxicity, but effectively promoted cell proliferation. The time-dependent proliferation of MOVAS cells treated with 25 mM glucose further confirmed this effect (Figure 2B). Treatment with Se-SP or SP did not cause cytotoxicity (Figure 2C). However, Se-SP co-treatment effectively inhibited the abnormal glucose-induced proliferation of MOVAS cells (Figure 2D). For instance, treatment with 5 and 10 μg/ml Se-SP significantly inhibited glucose-induced proliferation from 152.6% (glucose, 25 mM) to 121.2 and 112.6%, respectively. Pre-treatment of cells with 5 or10 μg/ml SP did not yield a significant protective effect. Taken together, the addition of Se afforded Se-SP enhanced potential to inhibit high glucose-induced proliferation of MOVAS cells.

### Se-PC Attenuates High Glucose-Induced Calcification of MOVAS Cells

**FIGURE 3:**
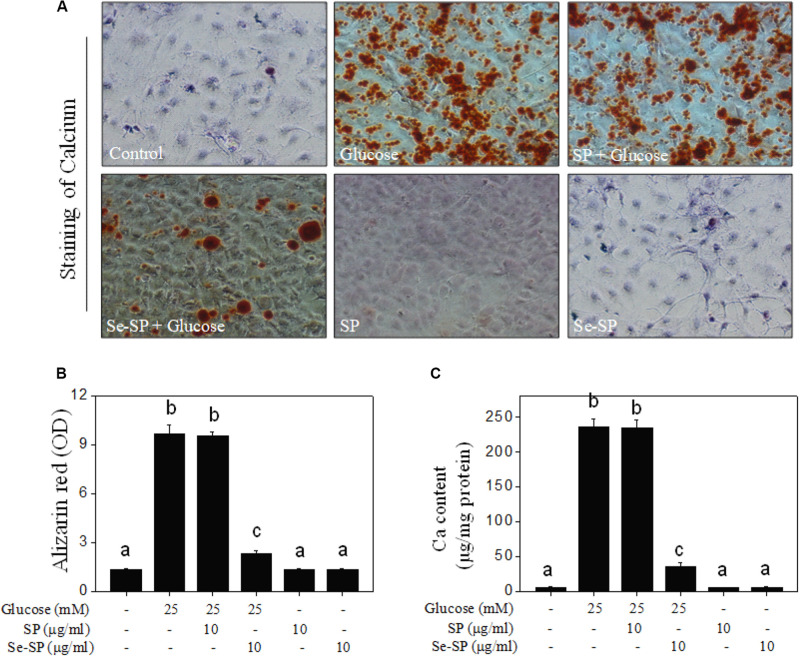
Se-SP attenuates high glucose-induced calcification of MOVAS cells. **(A)** Se-SP inhibited high glucose-induced calcification of MOVAS cells. MOVAS cells seeded in six-well plate were treated with 10 μg/ml Se-SP or/and 25 mM glucose for 14 days. Calcium nodules were stained by alizarin red and observed under light microscope. **(B)** Absorbance of alizarin red. Alizarin red dye in the parallel experiments was eluted with 10% formic acid and quantified by a microplate reader at 420 nm and expressed as the optical density units. **(C)** Determination of calcium content. Protein was quantified by BCA kit, and calcium level was measured by O-cresolphthalein complexone method. Calcium content was expressed as μg/mg protein. All data were shown from three different experiments. Bars with different letters are statistical significance at the level of *P* < 0.05.

It was reported that high glucose could induce VSMC calcification (Wang et al., 2019), so 25 mM glucose was used in this study. Cell calcification was examined by alizarin red staining, and the results showed that MOVAS cells exposed to 25 mM glucose showed significant calcification, as demonstrated by the increased numbers of deep red calcium nodules (Figure 3A). The absorbance of alizarin red eluted from calcified cells indirectly reflected glucose-induced calcification of MOVAS cells (Figure 3B). Moreover, quantitative analysis of calcium content confirmed glucose-induced calcification of MOVAS cells (Figure 3C). Notably, Se-SP co-treatment significantly attenuated glucose-induced calcification, as evidenced by the decreases in deep red calcium nodules, absorbance, and calcium content. Co-treatment with SP showed no significant protective effect. Taken together, our results suggest that Se-SP after Se addition achieved enhanced potential to attenuate high glucose-induced calcification of MOVAS cells.

### Se-SP Suppresses High Glucose-Induced ALP Activity and Expression

**FIGURE 4:**
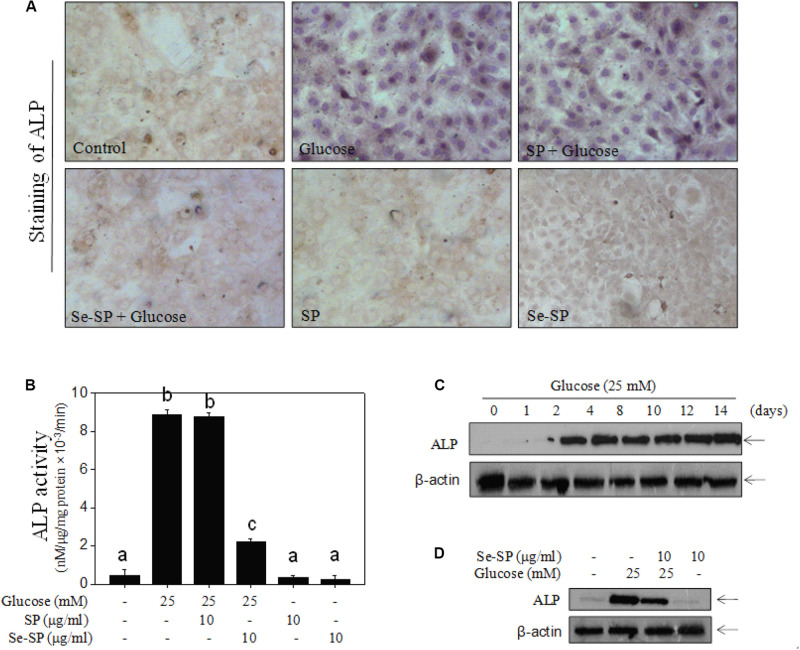
Se-SP suppresses high glucose-induced ALP activity and expression. **(A)** ALP staining of MOVAS cells. MOVAS cells seeded in six-well plate were co-treated with 10 μg/ml Se-SP or/and 25 mM glucose for 14 days. ALP staining was conducted by an ALP staining kit. **(B)** Determine of ALP activity of MOVAS cells. Total protein of cells was prepared and quantified by BCA kit. ALP activity was examined by an ALP activity kit according to the manufacturer’s instructions. **(C)** Time-dependent expression of ALP in glucose-treated MOVAS cells. MOVAS cells were treated with 25 mM glucose for 1–14 days. **(D)** Se-PC inhibits ALP expression in high glucose-treated cells. ALP expression was detected by western blot analysis.

Alkaline phosphatase is an early osteogenesis marker that is highly expressed in calcified VSMCs (Liu et al., 2013). Here, ALP activity and ALP expression were examined in MOVAS cells. As shown in Figure 4A, treatment with 25 mM glucose for 14 days caused ALP activation of ALP as detected by a staining kit. The results from ALP activity kits showed that glucose treatment obviously increased ALP activity (Figure 4B). This was confirmed by the time-dependent upregulation of ALP expression in glucose-treated cells (Figure 4C). However, Se-SP co-treatment significantly attenuated glucose-induced ALP activation, as shown by decreased ALP staining, activity, and expression (Figure 4D). Co-treatment with SP showed no significant protective effect. Taken together, the addition of Se imbued Se-SP with enhanced potential to suppress high glucose-induced ALP activation in MOVAS cells.

### Se-SP Prevents ROS-Mediated DNA Damage in Glucose-Treated MOVAS Cells

**FIGURE 5:**
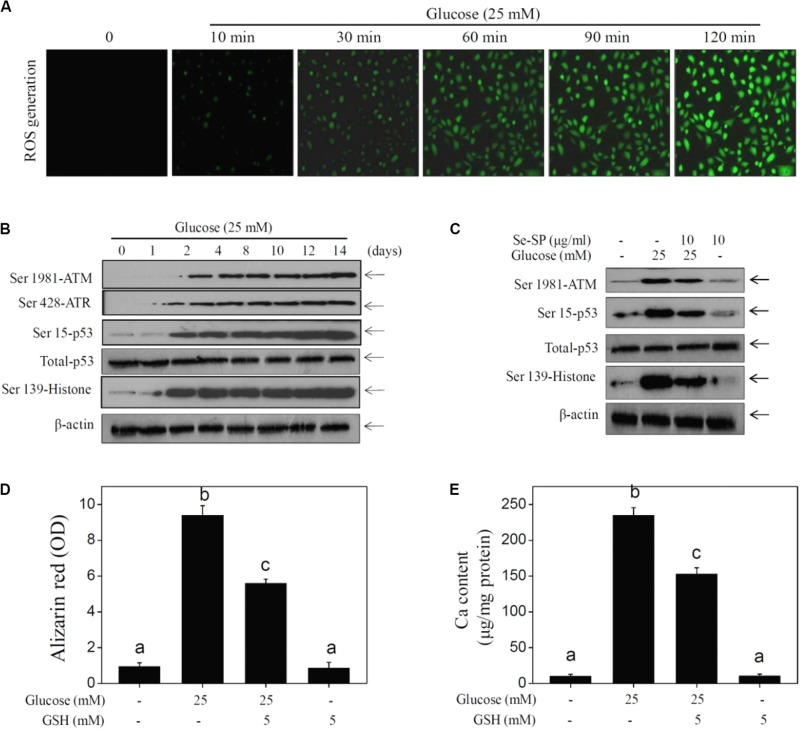
Se-SP prevents ROS-mediated DNA damage in glucose-treated MOVAS cells. **(A)** High glucose-induced time-dependent ROS generation. MOVAS cells seeded in six-well plate were treated with 25 mM glucose for 10–120 min. DCFH-DA probe was loaded and the ROS generation (green fluorescence) was imaged by a fluorescent microscope. **(B)** High glucose-induced time-dependent DNA damage. MOVAS cells seeded in 9-cm plate were exposed to 25 mM glucose for 1–14 days. **(C)** Se-PC prevents high glucose-induced DNA damage. MOVAS cells were treated with 10 μg/ml Se-SP or/and 25 mM glucose for 14 days. **(D)** Absorbance of alizarin red. Alizarin red dye in the parallel experiments was eluted with 10% formic acid and quantified by a microplate reader at 420 nm and expressed as the optical density units. **(E)** Determination of calcium content. MOVAS cells were pre-treated with 5 mM GSH for 2 h before glucose treatment. Protein was quantified by BCA kit, and calcium level was measured by O-cresolphthalein complexone method. Calcium content was expressed as μg/mg protein. Protein expression was examined by western blotting.

Accumulated evidence indicates that ROS-mediated oxidative stress can induce VC (Chen N. X. et al., 2006; Liu et al., 2014; Qiu et al., 2017). Hence, ROS generation and oxidative damage were both examined in calcified MOVAS cells using a DCFH-DA probe and DNA damage markers, respectively. As shown in Figure 5A, glucose treatment caused notable and time-dependent ROS generation (green fluorescence) that was observed as early as 10 min. Markers of DNA damage were detected by western blotting, and the results showed that glucose treatment significant caused DNA damage, as evidenced by the enhanced phosphorylation of ATM (Ser1981), ATR (Ser428), p53 (Ser15), and histones (Ser139) (Figure 5B). Se-SP co-treatment significantly decreased phosphorylation of all three proteins (Figure 5C). What is more, ROS inhibition by GSH effectively inhibited high glucose-induced calcification (Figures 5D,E), indicating that Se-SP could act as ROS inhibitor to inhibit high glucose-induced DNA damage and calcification. Taken together, these results indicate that Se-SP suppressed ROS-mediated DNA damage and calcification in glucose-treated MOVAS cells.

### Se-SP Improves High Glucose-Induced Dysfunction of MAPKs and PI3K/AKT Pathways

**FIGURE 6:**
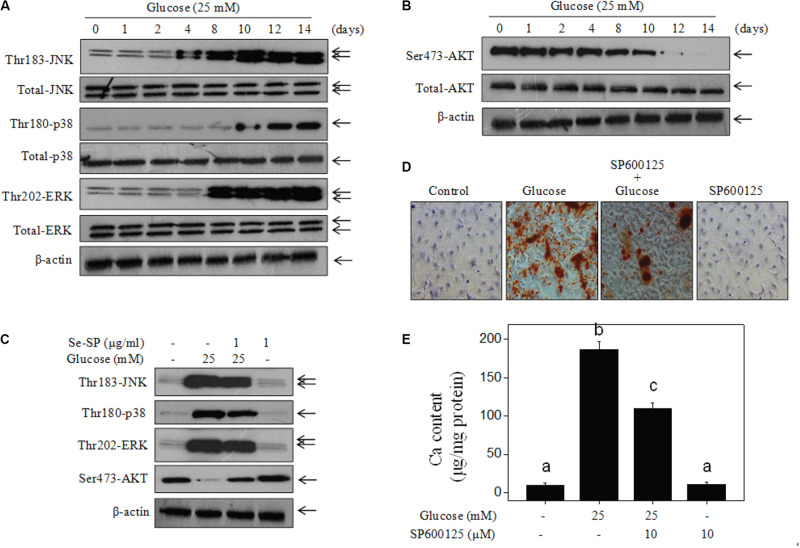
Se-SP improves high glucose-induced dysfunction of MAPKs and PI3K/AKT pathways. **(A)** High glucose-induced time-dependent activation of MAPKs pathway. **(B)** High glucose-induced time-dependent inactivation of PI3K/AKT pathway. MOVAS cells seeded in 9-cm plate were treated with 25 mM glucose for 1–14 days. **(C)** Se-SP improves high glucose-induced dysfunction of MAPKs and PI3K/AKT pathways. Western blotting was used to examine protein expression. **(D)** SP600125 (JNK inhibitor) blocked high glucose-induced calcification of MOVAS cells. MOVAS cells were co-treated with 10 μM SP600125 and 25 mM glucose for 14 days. Calcium nodules were stained by alizarin red. **(E)** Measurement of calcium content. Bars with different letters are statistical significance at the level of *P* < 0.05.

Studies have confirmed significant roles of MAPKs and PI3K/AKT in regulating VSMC calcification (Liu et al., 2010, 2014). Therefore, the status of MAPK and PI3K/AKT signaling pathway proteins in glucose-treated MOVAS cells was assessed by western blotting. The results showed that glucose treatment time-dependently triggered MAPK activation, as evidenced by the enhanced phosphorylation of p38 kinase, c-Jun N-terminal kinase (JNK), and extracellular regulated kinase (ERK) (Figure 6A). As expected, glucose treatment caused time-dependent inactivation of AKT (Figure 6B). However, Se-SP co-treatment significantly inhibited MAPK activation and AKT inactivation in glucose-treated MOVAS cells (Figure 6C). Moreover, inhibition of JNK (by SP600125) obviously blocked high glucose-induced calcification of MOVAS cells (Figures 6D,E), which further confirmed the significance of MAPK signaling. Taken together, these results indicated that Se-SP has the potential to attenuate high glucose-induced dysfunction in MAPK and PI3K/AKT signaling.

## Discussion

Chronic hyperglycemia is the main characteristic of diabetes, which can cause vascular inflammation, vasoconstriction, thrombosis, VC, and atherosclerosis by stimulating ROS overproduction and generation of pro-inflammatory cytokines and advanced glycation end products (Paneni et al., 2013; Baktiroglu et al., 2016; Henning, 2018). Cerebral arterial calcification is associated with the occurrence of stroke (Bugnicourt et al., 2011), cognitive impairment, and dementia (Bos et al., 2015), and a high level of cerebral arterial calcification is emerging as a predictor of poor neurological recovery after revascularization treatment in ischemic stroke patients (Lee et al., 2015). Studies have demonstrated that Se can inhibit VSMCs calcification by attenuating oxidative stress (Liu et al., 2010, 2014). However, the protective effect and mechanism of Se-containing protein against VSMC calcification has not been reported yet.

High glucose is a typical hallmark of diabetes and is involved in atherosclerosis and VC (Baktiroglu et al., 2016), contributing to the high morbidity and mortality of CCD (Bugnicourt et al., 2011; Leon and Maddox, 2015). VSMCs play an important role in the process of diabetic VC. Calcified VSMCs exhibited increased migration, proliferation, and apoptosis (He et al., 2012; Shi et al., 2017). Previous findings reported that high glucose conditions could induce calcification and osteogenic transformation (Chen N. X. et al., 2006; Shi et al., 2017). The present study also showed that high glucose (25 mM, 48 h) effectively induced abnormal proliferation of MOVAS cells and induced significant calcification (25 mM, 14 days), which was consist with previous findings (Chen N. X. et al., 2006; He et al., 2012; Shi et al., 2017). The results also confirmed that abnormal proliferation was necessary for VC development (Durham et al., 2018).

Oxidative stress is critical mediator in the progression of diabetic atherosclerosis and VC (Matough et al., 2012; Carrillo-Sepulveda et al., 2015; Baktiroglu et al., 2016). Organisms produce numerous ROS in normal metabolism, including hydrogen peroxide, hydroxyl radicals, and superoxide anions. Meanwhile, organisms possess various antioxidant systems to scavenge excessive ROS, such as superoxide dismutase, GSH peroxidase, and peroxidase reductase. Long-term uncontrolled high glucose in diabetes may destroy the redox balance and cause oxidative damage to biological macromolecules and cells (Matough et al., 2012), including lipid peroxidation, protein denaturation, DNA breakage, and cell death. However, apoptotic bodies produced by late apoptotic VSMCs can serve as an ucleation structure for calcium crystals and induce VC (Shi et al., 2015, 2017). The present findings revealed that high glucose induced significant ROS generation and subsequent DNA damage, which contributed to high glucose-induced calcification of MOVAS cells.

The MAPK family composed of p38 kinase, ERK, and stress-activated protein kinase (SAPK)/JNK can transmit extracellular stimulation signals to the cell nucleus and regulate diversified biological functions including cell proliferation, differentiation, and apoptosis (Zhang et al., 2019). PI3K/AKT is an important intracellular signaling pathway regulating cell survival and apoptosis. A recent report provided evidence that high glucose can stimulate VC by activating different signal transduction pathways in VSMCs. Shi et al. (2017) showed that high glucose spurred alterations in VSMC migration, proliferation, calcification, and apoptosis via the upregulation of ERK1/2 phosphorylation, resulting in VC. Chen N. X. et al. (2006) revealed that high glucose induced VC by increasing the expression of bone-related molecules (BMP-2, Cbfa1) via activation of the protein kinase C pathway. Furthermore, ROS play a critical role in MAPK and PI3K/AKT signaling in diabetes. Li et al. (2013) found that ROS induced by high glucose initiated VSMC calcification by activating MAPK, PI3K/AKT, and nuclear factor (NF)-kB signaling proteins. Su et al. (2019) reported that high glucose induced VSMCs proliferation, adhesion, and migration through activation of the ERK pathway mediated by ROS. Our findings revealed that exposure of VSMCs to high glucose promoted MAPK activation and PI3K/AKT inactivation, which confirmed their significance of in VC.

Se has novel antioxidant functions and is an important and essential micronutrient for human health (Rayman, 2000). *S. platensis* is a widely used health food discovered to be a suitable carrier for Se accumulation, and increasing evidence demonstrates that Se-SP has stronger antioxidant activities than the protein without Se derived from *S. platensis* (Chen and Wong, 2008; Fan et al., 2012; Shi et al., 2017). The present study demonstrated that Se-PC co-treatment of MOVAS cells exerted protective effects against high glucose-induced proliferation and calcification, inhibited ALP and MAPK activation, inactivated AKT, and decreased ROS-mediated DNA damage. The addition of Se to Se-SP increased the potential to inhibit high glucose-induced calcification of MOVAS cells through inhibiting ROS-mediated DNA damage and regulating MAPK and AKT signaling.

## Data Availability Statement

The original contributions presented in the study are included in the article/supplementary material. Further inquiries can be directed to the corresponding author/s.

## Ethics Statement

This manuscript contained no animal experiments and patients’ experiments. Therefore, there was no ethical standard to be declared.

## Author Contributions

WC and PZ conceived the project and designed this work. CL, LZ, BL, FZ, QS, GK, XW, SC, and RD performed the experiments. CL and LZ wrote the manuscript. All authors analyzed the data and revised the manuscript.

## Conflict of Interest

The authors declare that the research was conducted in the absence of any commercial or financial relationships that could be construed as a potential conflict of interest.
